# Maximal fat oxidation rate across the adult lifespan of trained women

**DOI:** 10.1002/ejsc.12027

**Published:** 2024-01-30

**Authors:** I. M. Dahlgaard Hansen, J. F. Wismann, R. E. Sahl, J. Frandsen, M. Hansen, A. Ingersen, M. Schmücker, J. L. Modvig, F. Dela, S. Larsen, J. W. Helge

**Affiliations:** ^1^ Xlab Center for Healthy Aging Department of Biomedical Sciences Faculty of Health and Medical Sciences University of Copenhagen Copenhagen Denmark; ^2^ Department of Geriatrics Bispebjerg‐Frederiksberg University Hospital Copenhagen Denmark; ^3^ Clinical Research Centre Medical University of Bialystok Bialystok Poland

**Keywords:** aging, fat oxidation, women

## Abstract

The fat oxidation capacity is higher in young compared to elderly subjects and higher in premenopausal compared to postmenopausal women, but the influence of age on maximal fat oxidation (MFO) is not clear. Therefore, this study aimed to evaluate MFO (g/min) across the lifespan of trained adult women. In total, 36 healthy trained women were recruited into three groups: (*n* = 12), young (27 ± 3 years, mean ± SD) premenopausal, middle‐aged (57 ± 3 years), and older (71 ± 2 years) postmenopausal women and all had a body mass index <25 kg/m^2^. After an overnight fast, body composition was determined by dual‐energy X‐ray absorptiometry, and blood samples were obtained. A FAT_max_‐test was performed on a cycle ergometer, and MFO was calculated from the pulmonary V̇O_2_ and V̇CO_2_ measured by indirect calorimetry. The absolute MFO was significantly higher in young (0.40 ± 0.07 g/min) compared to both middle‐aged (0.33 ± 0.07 g/min) (*p* = 0.035) and old (0.25 ± 0.05 g/min) women (*p* < 0.001). Absolute MFO was higher in middle‐aged compared to old women (*p* = 0.018). Relative MFO (MFO/LBM, mg/min/LBM) was higher in young (8.39 ± 1.62 mg/min/LBM) compared to old (6.16 ± 1.14 mg/min/LBM) women (*p* = 0.004). A significant linear relationship was observed between absolute MFO and age (*R* ^2^ = 0.41; *p* < 0.001), V̇O_2max_ (*R* ^2^ = 0.40; *p* < 0.001), and LBM (*R* ^2^ = 0.13; *p* = 0.033), respectively, and between relative MFO and fat mass (*R* ^2^ = 0.12; *p* = 0.04). In conclusion, the maximal capacity to oxidize fat is attenuated with age in trained women. Furthermore, postmenopausal middle‐aged women have higher absolute MFO compared to older women, and this implies that it is age per se and not a change in estrogen availability that leads to lower absolute MFO.

## Highlights

- In trained women, the maximal fat oxidation was lowered with age when comparing young, middle‐aged, and older groups implying an attenuating effect of age.
- In postmenopausal women, the maximal fat oxidation was lower in older than middle‐aged groups, and this implies that age and not estrogen availability leads to a lower absolute maximal fat oxidation rate.
- In trained women, the FAT_max_ was similar when comparing young, middle‐aged, and older groups.

**Abbreviations**
BMI: body mass index
DXA: dual‐energy X‐ray absorptiometry
FFA: free fatty acids
HDL: high‐density lipoprotein
LBM: lean body mass
LDL: low‐density lipoprotein
MFO: maximal fat oxidation
RER: respiratory exchange ratio
TG: triacylglycerol

## 1 INTRODUCTION

Aging is associated with changes in several physiological parameters, including a reduction in maximal oxygen uptake (V̇O_2max_), lean body mass (LBM), muscle strength (Forbes et al., 1970; Hawkins et al., 2003; McGavock et al., 2009), and an increase in fat mass and visceral fat (Lovejoy et al., 2008). The physiological changes that occur with aging could be due to a decrease in daily physical activity and/or aging per se (Shur et al., 2021). Only a few studies have investigated the capacity to use and oxidize fat in relation to aging. In a cross‐sectional study, it was observed that elderly subjects (73 ± 2 years) had 25%–35% lower fat oxidation during 60 min of moderate‐intensity exercise than young subjects (26 ± 2 years) at both the same absolute (0.81 ± 0.1 and 0.84 ± 0.09 mL O_2_/min, respectively) and relative (55.7 ± 3.1 and 50.6 ± 1.3% VO_2max_, respectively) intensity (Sial et al., 1996). In the same study, lipolytic rates and fatty acid (FA) availability were not rate‐limiting in older subjects, and the authors suggested that the reduction in fat oxidation during moderate‐intensity exercise was caused by age‐related attenuation of skeletal muscle mitochondrial volume and thus respiratory capacity (Sial et al., 1996). Using a different approach Lovejoy et al. (2008) found that the 24‐h fat oxidation rate was reduced by 32% in women who were premenopausal at baseline but postmenopausal at follow‐up (4 years later), and interestingly, the 24‐h fat oxidation was unchanged in women who remained premenopausal at follow‐up (Lovejoy et al., 2008). In line with this, a cross‐sectional study of age‐matched women reported that fat oxidation rate during 45 min of exercise at 50% of V̇O_2max_ was 33% lower in postmenopausal women (52.0 ± 2.0 years) than premenopausal women (50.1 ± 2.1 years) (Abildgaard et al., 2013). Based on these findings, it appears that a reduction in fat oxidation rate is associated with the hormonal changes at menopause, markedly decreased estrogen, progesterone, and testosterone concentrations per se and not aging (Abildgaard et al., 2013; Lovejoy et al., 2008).

During submaximal exercise, lipids are the primary supply of energy, and across the last decade, major interest has been directed toward the role and regulation of maximal fat oxidation (MFO; g/min) and the exercise intensity at which MFO occurs (FAT_max_; % V̇O_2max_) (Achten et al., 2002). It is well known that the MFO rate depends on various factors such as training status, sex, LBM, and nutritional status (Maunder et al., 2018; Purdom et al., 2018). In a recent study, a higher MFO rate was observed in trained than untrained women both in young and middle‐aged postmenopausal women (Frandsen, Hansen, et al., 2021). Interestingly, a similar MFO rate was observed in a large cohort (*N* = 188 women) study when comparing below and above average V̇O_2max_ groups in peri‐ and postmenopausal women, and a lower relative MFO (MFO/LBM) rate was only observed when postmenopausal and young women were compared (Frandsen, Amaro‐Gahete, et al., 2021).

There is, to the best of our knowledge, no data on MFO rate in older women (>65 years). Based on this, the present study aimed to apply a cross‐sectional design to investigate MFO rates across the trained adult woman's lifespan. Based on the existing literature, we hypothesized that the MFO rate would be reduced with age in trained women.

## 2 METHODS

### 2.1 Participants

This study recruited 36 healthy trained women and classified them into three groups: young (*n* = 12, age: 18–35 years), middle‐aged (*n* = 12, age: 55–65 years), and old (*n* = 12, age: 70–80 years). The middle‐aged and older women were all postmenopausal (>12 months since the last menstrual period). The women were eligible for participation if they had a body mass index (BMI) <25 kg/m^2^, practiced endurance training weekly, and had a maximal oxygen uptake above; young women: V̇O_2max_ > 50 mL/min/kg, middle‐aged women: V̇O_2max_ > 40 mL/min/kg, and old women: V̇O_2max_ > 20 mL/min/kg. The selection of these V̇O_2max_ levels was based on a large Danish Cohort study that measured cardiorespiratory fitness levels in a large Danish representative sample (Eriksen et al., 2016). Furthermore, the women were required not to have any medical condition or use medication known to affect metabolism (i.e., glucose and fat oxidation) or estrogen concentration. All women were nonsmokers.

The data and results from two of the groups, the young and middle‐aged women, have been published in a former publication (Frandsen, Hansen, et al., 2021), however, with a different focus and aim.

Participants received oral and written information regarding the study and had the possibility of at least 48 h of consideration before signing the consent form. The study was approved by the research ethics committee of the greater region of Copenhagen (H‐19034328) and adhered to the principles of the Helsinki declaration.

### 2.2 General design

The participants were told to refrain from vigorous physical activity and exercise, avoid alcohol, and eat their normal habitual diet 24 h before the test. Participants were further instructed to eat no later than 22:00 h in the evening before the second test and to commute to the laboratory by car or public transportation. All the exercise tests were conducted in the morning between 07:00 and 09:00 h after an overnight fast, lasting from 22.00 in the evening before providing a minimum of 9 h fasting.

Participants came to the laboratory on two separate occasions (separated by 3–14 days). At the first visit, a brief interview regarding their health, habitual diet, training habits, and medical history was performed. Hereafter, BMI was calculated and an incremental maximal exercise test (V̇O_2max_‐test) was performed to establish the training state of the participants. At the second visit, height and body weight were measured after which a dual‐energy X‐ray absorptiometry scan (Lunar Prodigy Advance, Lunar Madison) was conducted to measure body composition. Visceral fat content was estimated from the DXA scan using the Core Scan software (GE Health Care). A venous blood sample was then collected from an antecubital vein. Subsequently, a graded submaximal exercise test was performed, followed by a 5‐min rest period before a maximal incremental exercise V̇O_2max_ test was conducted. Lastly, the subject's handgrip and leg extension strength were measured with a hand dynamometer (Takei Scientific Instruments, T.K.K. 5401) and with a Nottingham Power Leg Rig 3.0, respectively. For each strength measure, three measurements separated by 1 min were performed and the highest measured results were chosen.

### 2.3 Experimental design

#### 2.3.1 Blood samples

Blood samples were collected in precooled vacutainers (Vacutainer BD) and immediately centrifuged at 4000 rpm for 10 min at 4°C (Centrifuge Hettich Universal 30 RF; Hettich), and the plasma fraction was stored at −80°C for later analysis (COBAS 6000 analyzer 501C; Roche). The plasma samples were analyzed for metabolites (i.e., glucose, free fatty acids [FFA], lactate, and glycerol), hormones (i.e., insulin, progesterone, and estradiol), and lipids (i.e., triacylglycerol [TG], low‐density lipoprotein [LDL] cholesterol and high‐density lipoprotein [HDL] cholesterol). In addition, HbA1c% was measured (DCA 2000+; Bayer Healthcare). HOMA‐IR was calculated as [fasting glucose (mmol/L) × fasting insulin (mU/mL)]/22.5 (Matthews et al., 1985).

#### 2.3.2 Exercise tests

Exercise tests were performed on a cycle ergometer (Monarch E839). The submaximal graded exercise tests conducted in this study were previously validated (Achten et al., 2002; Dandanell et al., 2017). The submaximal graded exercise test was initiated with a 5‐min seated rest on the cycle ergometer. Then, both the young and middle‐aged women started cycling at 35 W with a 25‐W increase every 3 min. The older women, however, started cycling at 15 W with a 15‐W increase every 3 min. When the participants reached a 30‐s period with a respiratory exchange ratio (RER) >1.0, no further workload was applied. The graded exercise protocol was adapted group‐wise to accommodate the differences in V̇O_2max_ among the young, middle‐aged, and old groups (i.e., to achieve a sufficient amount of data points).

The V̇O_2max‐_test protocol was similar between the first and second test days. The young women initiated the test at a workload of 150 W, the middle‐aged group at 120 W, and the older group at 80 W. For all participants, 20 W were applied every minute until voluntary exhaustion, and all were verbally encouraged to continue until exhaustion. V̇O_2max_ was accepted when two of the following three criteria were met: (i) a leveling off in V̇O_2_ (<2.1 mL/min/kg) despite increased workload, (ii) an RER >1.15 for the last 30 s of the test, (iii) or achievement of a maximal heart rate (±5 bpm, 220‐age).

Pulmonary V̇O_2_ and V̇CO_2_ were measured breath‐by‐breath with an automated online system (Oxycon Pro system, Jaeger). The gas analyzers were carefully calibrated with a 5% CO_2_–16% O_2_ in N_2_ gas mixture and an automated volume calibration before each exercise test. Substrate oxidation was calculated using the equation of Frayn with the assumption that the urinary nitrogen excretion rate was negligible (Frayn, 1983).

#### 2.3.3 Statistics and calculations

A third‐degree polynomial regression analysis was performed based on the calculated fat oxidation data for each participant. The highest value detected by the regression analyses and the relative intensity (% VO_2max_) at which it occurred was collected, and the mean values are reported in Tables 1 and 2. Figures showing third‐degree regression analyses are depicted as the mean fat oxidation rates (from the regression analyses) at 10%, 20%, 30%, 40%, 50%, 60%, 70%, and 80% of V̇O_2max_ and the subsequent third‐degree regression analysis on these mean values. One‐way analysis of variance was performed among the young, middle‐aged, and old women on the anthropometric data and data from plasma samples. If data were not normally distributed, they were log‐transformed. Linear regression analyses were performed to investigate the relationships between MFO as the dependent variable and age, estrogen, progesterone, leg strength, hand grip strength, and relative VO_2max_ as the independent variables. All data analyses and figures were made using Graphpad prism 8.0 (GraphPad Software).

**TABLE 1:** Subject characteristics.

	Young (*n* = 12)	Middle‐aged (*n* = 12)	Old (*n* = 12)	*p*‐value
Age, year	27 ± 3***^,†††^	58 ± 3^###^	71 ± 2	<0.001
Height, cm	168 ± 6	166 ± 3	166 ± 6	0.47
Weight, kg	60.8 ± 4.4	61.6 ± 4.8	62.8 ± 6.6	0.68
BMI kg/m^2^	21.5 ± 1.3	22.3 ± 1.8	22.8 ± 1.9	0.15
Fat mass, kg	13.7 ± 2.2^†††^	16.1 ± 3.0^###^	21.5 ± 4.3	<0.001
Fat mass, %	23.06 ± 2.70	26.48 ± 4.10	34.00 ± 4.95	0.21
Lean body mass, kg	47.3 ± 3.3^††^	45.7 ± 3.5^#^	41.3 ± 4.7	<0.01
Visceral fat, g	72 ± 70***^,†††^	260 ± 104	572 ± 378	<0.001
V̇O_2max_, mL/min	3115 ± 266***^,†††^	2506 ± 227^###^	1762 ± 283	<0.001
V̇O_2max_, mL/min/kg	51.4 ± 3.0***^,†††^	40.7 ± 2.2^###^	28.2 ± 4.4	<0.001
V̇O_2max_, mL/min/LBM	65.8 ± 4.0***^,†††^	54.9 ± 4.1^###^	42.6 ± 4.4	<0.001
Hand grip, kg	30.3 ± 5.0^††^	28.5 ± 4.1^#^	23.8 ± 3.6	<0.01
Leg strength, W/kg	2.7 ± 0.5^†††^	2.4 ± 0.4^###^	1.5 ± 0.5	<0.001

*Note*: Data are mean (±SD). *p* values indicate the overall result of one‐way analysis of variance and *, #, † shows the result of Tukey's post hoc test.

Abbreviation: BMI, body mass index.

*Significant difference from middle‐aged: *p* < 0.05, **p < 0.01, and ***p < 0.001.

^#^Significant difference from old: *p* < 0.05, ^##^ *p* < 0.01, and ^###^ *p* < 0.001.

^†^Significant difference from old: *p* < 0.05, ^††^ *p* < 0.01, and ^†††^ *p* < 0.001.

**TABLE 2:** Plasma concentrations at rest.

	Young (*n* = 12)	Middle‐aged (*n* = 12)	Old (*n* = 12)	*p*‐value
Triglyceride, mmol/L	0.67 ± 0.34	0.81 ± 0.37	0.91 ± 0.25	0.064
Total cholesterol, mmol/L	4.05 ± 0.62**^,††^	5.31 ± 0.80	5.22 ± 1.10	0.002
HDL, mmol/L	2.01 ± 0.33	2.26 ± 0.42^#^	1.89 ± 0.22	0.03
LDL, mmol/L	2.13 ± 0.41*^,††^	3.10 ± 0.66	3.27 ± 1.10	0.002
Progesterone, nmol/L	4.09 ± 9.95^†^	0.82 ± 0.21	0.71 ± 0.28	0.20
Estrogen, pmol/L	180 ± 110***^,†††^	41 ± 11	45 ± 9	<0.001
Insulin, pmol/L	34.0 ± 11.6	29.5 ± 10.3	36.9 ± 12.5	0.28
Glycerol, μmol/L	57.7 ± 45.0	53.1 ± 32.0	45 ± 13.7	0.63
FFA, μmol/L	472 ± 318	471 ± 170	441 ± 124	0.93
Glucose, mmol/L	4.98 ± 0.38	5.23 ± 0.33	5.32 ± 0.46	0.11
HbA1c, %	5.00 ± 0.59	5.30 ± 0.61	5.39 ± 0.23	0.18
HOMA‐IR	1.10 ± 0.45	0.99 ± 0.37	1.28 ± 0.51	0.29

*Note*: Data are mean (±SD). *p* values indicate an overall result of one‐way analysis of variance and *, #, † indicate the result of Tukey's post hoc test.

Abbreviations: FFA, free fatty acids; HDL, high‐density lipoprotein; LDL, low‐density lipoprotein.

*Significant difference from middle‐aged: *p* < 0.05, **p < 0.01, and ***p < 0.001.

^#^Significant difference from old: *p* < 0.05, ^##^ *p* < 0.01, and ^###^ *p* < 0.001.

^†^Significant difference from old: *p* < 0.05, ^††^ *p* < 0.01, and ^†††^ *p* < 0.001.

## 3 RESULTS

The present study and dataset is a secondary analysis, and as such, an individual, separate power calculation was not performed. The primary analysis has been published in a separate paper (Frandsen, Hansen, et al., 2021).

### 3.1 Subject characteristics

The three groups were similar in BMI, body weight, and height (Table 1). The young and middle‐aged women had significantly lower fat mass compared to the old women (Table 1). Furthermore, the LBM was significantly higher in both the young (*p* < 0.01) and the middle‐aged women (*p* < 0.05) when compared to the old women (Table 1). There were no significant differences in fat mass and LBM between the young and middle‐aged women (Table 1). The absolute and relative (both per total body weight and LBM) V̇O_2max_ were, as expected, significantly higher in young women compared to both middle‐aged and old women (*p* < 0.001) (Table 1). Absolute and relative (both per total weight and LBM) V̇O_2max_ were also significantly higher in middle‐aged women compared with old women (*p* < 0.001) (Table 1). Grip and leg strength were significantly higher in young women compared to middle‐aged and older women and the middle‐aged women's grip and leg strength were higher than the older women (Table 1).

### 3.2 Maximal fat oxidation rate and FAT_max_

**FIGURE 1:**
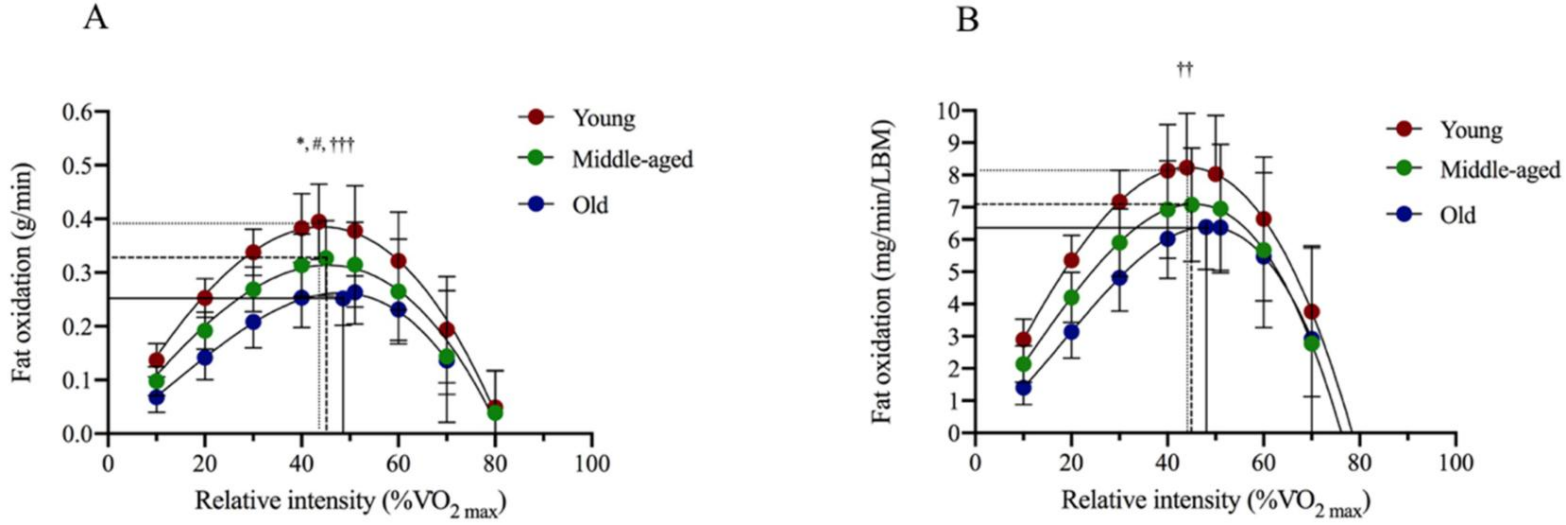
Maximal fat oxidation rates expressed as absolute (A) and relative to lean body mass (B) in young, middle‐aged, and old women. The lines are third‐order polynomial regression fit of mean ± SD fat oxidations rate at 20%, 30%, 40%, 50%, 60%, 70%, and 80% of V̇O_2max_. Horizontal lines indicate MFO and vertical lines indicate FAT_max_. The MFO rate and FAT_max_ are illustrated by horizontal and vertical lines. *Significant difference between young and middle‐aged: *p* < 0.05, **p < 0.01, and ***p < 0.001. ^#^Significant difference between middle‐aged and old: *p* < 0.05, ^##^ *p* < 0.01, and ^###^ *p* < 0.001. ^†^Significant difference between young and old: *p* < 0.05, ^††^ *p* < 0.01, and ^†††^ *p* < 0.001. MFO, maximal fat oxidation.

The absolute MFO (g/min) was significantly higher in young women compared to both middle‐aged (*p* = 0.035) and old women (*p* < 0.001). Absolute MFO (g/min) was also higher in middle‐aged compared to old women (*p* = 0.018) (Figure 1A). The relative MFO (mg/min/LBM) was higher in the young compared to the old women (*p* = 0.004) (Figure 1B), but there was no significant difference between middle‐aged and both young and old women, respectively. Furthermore, there was no significant difference in FAT_max_ (% VO_2max_) between the three groups.

### 3.3 Plasma hormones and metabolites at rest

Plasma estrogen and progesterone concentrations were significantly higher in young women compared to middle‐aged and old women (*p* < 0.001 and *p* < 0.01, respectively) (Table 2). The lipid profiles were different between the three groups. The plasma concentrations of total cholesterol were significantly lower in the young compared to the middle‐aged and older women (*p* < 0.001) (Table 2). The plasma concentration of HDL was higher in middle‐aged compared to old women (*p* < 0.05) (Table 2). The plasma concentration of LDL was significantly lower in the young compared to the middle‐aged and older women (*p* < 0.05 and *p* < 0.01, respectively) (Table 2). Although not statistically significant, a trend (*p* = 0.052) toward a lower concentration of plasma triglyceride (TG) was observed in the young compared to the old women. The plasma insulin, glucose, glycerol, and FFA concentrations were not different between the groups (Table 2). There was no significant difference in either HbA1c or HOMA‐IR between the three groups (Table 2).

### 3.4 Factors associated with MFO

**FIGURE 2:**
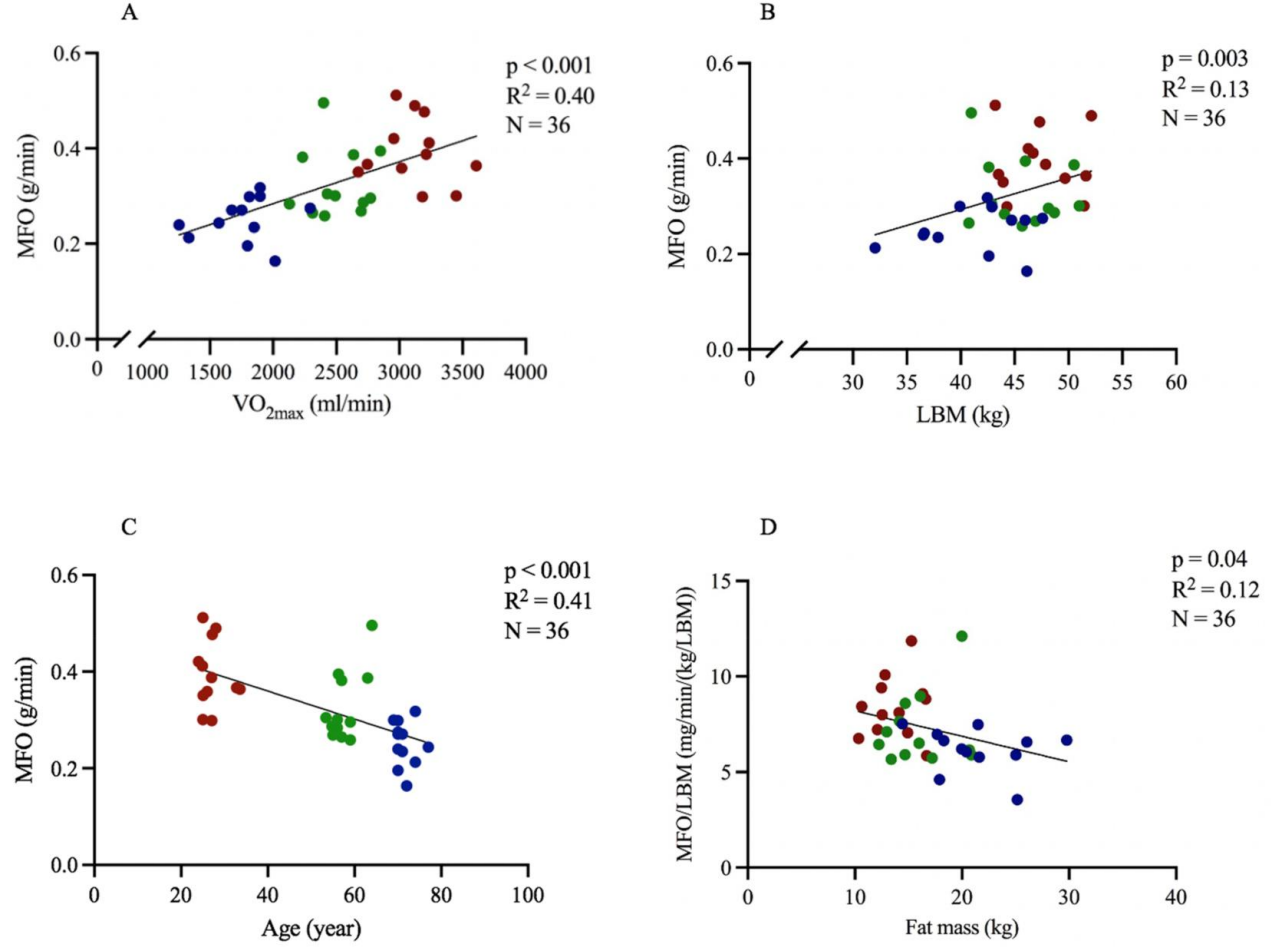
Simple linear regression of VO_2max_ (A), LBM (B), age (C), and fat mass (D) with the MFO rate or MFO/LBM rate as the dependent variable in 36 trained women (blue: old, green: middle‐aged, and red: young). LBM, lean body mass; MFO, maximal fat oxidation.

Simple linear regression analysis showed a significant linear relationship between the absolute MFO rate as the dependent and independent variables: V̇O_2max_ (*R* ^2^ = 0.40; *p* < 0.001), LBM (*R* ^2^ = 0.13; *p* = 0.033), age (*R* ^2^ = 0.41; *p* < 0.001) (Figure 2A–C), and leg strength (*R* ^2^ = 0.37; *p* < 0.001). Furthermore, there was a significant linear relationship between the MFO/LBM rate and fat mass (*R* ^2^ = 0.12; *p* = 0.04) (Figure 2D). There were no significant associations between absolute MFO rate and plasma FFA (*R* ^2^ = 0.004; *p* = 0.705) as well as plasma–estrogen concentrations (*R* ^2^ = 0.08; *p* = 0.098).

## 4 DISCUSSION

A major finding in this cross‐sectional study was that the MFO rate was lower with age in trained women, which supports our hypothesis. The lower MFO rate observed in old compared to middle‐aged women occurred without a change in estrogen exposure, indicating that the decreased aerobic fitness and lean mass with increasing age in the three groups are probably the major drivers. The second major finding was that the relative MFO rate (i.e., per LBM) was different between young and old women but not between middle‐aged and older women. The third major finding was that FAT_max_ remained unchanged with age in trained women.

### 4.1 Absolute MFO rate and VO_2max_

In line with our hypothesis, we observed that the absolute MFO rate was lowered with age in the three BMI and fitness level matched groups. The absolute MFO rate is closely related to fitness level as indicated by the correlation between V̇O_2max_ and MFO rate (Figure 2A), and as previously demonstrated in the literature (Frandsen et al., 2017; Venables et al., 2005). We recruited trained women with cardiorespiratory fitness levels well above average within their age group when compared to a large Danish representative sample (Eriksen et al., 2016). Most longitudinal and cross‐sectional studies to date demonstrate that V̇O_2max_ peaks when subjects are in their twenties and that V̇O_2max_ declines approximately 10% per decade (Hawkins et al., 2003). However, McGavock et al. (2009) showed a nonlinear decline in V̇O_2max_ in men, where the reduction was greater in the 10 years from age 50 to 60 compared to the previous 30‐year interval from age 20 to 50 (−17% vs. −8%) (McGavock et al., 2009). In the present study, V̇O_2max_ was 30% lower in older women compared to middle‐aged women (−2.1% per year) and 20% lower in middle‐aged women compared to young women (−1.5% per year), and although the study is cross‐sectional in design, this falls in line with the above finding in men.

### 4.2 Relative MFO and respiratory capacity

Our second major finding was that the MFO/LBM rate was lower in old women compared to young women, but there was no difference in MFO/LBM rate when young and middle‐aged as well as old and middle‐aged women were compared. In addition to age‐related structural changes in cardiovascular function (Kane et al., 2018), other physiological factors regulating fat oxidation may occur with age, and thus influence the MFO rate. Abildgaard et al. (2013) found that LBM correlated closely with the whole body fat oxidation in pre‐, peri‐, and postmenopausal women that only differed in mean age by 3 years (Abildgaard et al., 2013). In the present study, the LBM was similar in young and middle‐aged women, and this may, at least partly, explain the similar MFO/LBM rate despite the difference in menopause status. In a previous study, a significantly lower mitochondrial respiratory capacity per mitochondrion was observed in middle‐aged compared to young men, despite that, they were matched for V̇O_2max_ (Larsen et al., 2012). This implies that the mitochondrial oxidative capacity per mitochondrion declines with age and together with a lower V̇O_2max,_ it may contribute to the significant reduction in the MFO/LBM rate from young to old women observed in this study. This finding is in line with Sial et al. (1996) who suggested that age‐related changes in skeletal muscle respiratory capacity probably mediated the observed reduction in whole‐body submaximal fat oxidation when comparing young and old gender and LBM‐matched subjects (Sial et al., 1996).

### 4.3 Age‐related increase in fat mass

In the present study, we observed a higher fat mass and visceral fat content in old women compared to both young and middle‐aged women, despite similar BMI and recruitment based on an age‐matched relative fitness level (Table 1). Interestingly, a weak significant positive correlation between MFO/LBM rate and fat mass was observed in this study, which is in line with studies indicating a higher MFO rate in obese compared to lean subjects when matched for aerobic fitness (Amaro‐Gahete et al., 2019; Ara et al., 2011). We have previously demonstrated a very strong, positive association between plasma FFA and MFO in both well‐trained men (Frandsen et al., 1985) and women (Frandsen, Poggi, et al., 2021). However, there is some controversy about the association between fat mass and plasma FFA. Some studies find a positive correlation (Boden, 2008; Mittendorfer et al., 2009) and others suggest that there is no direct association (Karpe et al., 2011). Albeit the age difference is a factor in this study, the higher fat mass in the old group was not accompanied by a higher plasma FFA at rest in the older women compared to middle‐aged and young women, which is in line with the observation of a lower MFO rate in the older group.

Prior studies found that fat oxidation was lower during exercise in women across menopause, but this was not directly coupled to the changes in plasma estrogen levels (Abildgaard et al., 2013; Lovejoy et al., 2008). In line with this, the present study found no correlation between plasma estrogen levels and the MFO rate, potentially because two of the three groups were postmenopausal women with low plasma estrogen levels. Interestingly, the observation of a lower absolute MFO rate in old compared to middle‐aged women implies that it is aging per se that lowers the MFO rate in postmenopausal women.

### 4.4 Age, training, and FAT_max_

There is evidence from cross‐sectional (Nordby et al., 2006) (Frandsen et al., 2020) and longitudinal training studies in men (Nordby et al., 2015; Rosenkilde et al., 2013) that FAT_max_ is higher with improved training status. However, in women, a cross‐sectional study found no difference in FAT_max_ between untrained and trained women (Frandsen, Hansen, et al., 2021). In the latter study, we also observed no age effect on FAT_max_ when comparing young and middle‐aged fit and unfit women (Frandsen, Hansen, et al., 2021), and here, we extend these findings and demonstrate that FAT_max_ remained unchanged with age also in older trained women. We are not aware of other studies that focus on the potential effect of age on FAT_max_ in women, but we have previously found that in men, there was a lower FAT_max_ in trained middle‐aged compared to young, trained men (Frandsen et al., 2020). Venables and colleagues demonstrated in a very large sample that FAT_max_ was lower in men compared to women (Venables et al., 2005), but it is not clear if sex difference is important for the lack of an effect of age on FAT_max_ in women. In this study, we focused on the effect of age in trained women to minimize the confounding effect that is a consequence of aging being an effect of both decreased physical activity (inactivity) and age per se (Shur et al., 2021).

### 4.5 Limitations

Despite the well‐matched groups (BMI and relative fitness level), the design of our cross‐sectional study has the limitation of causal interpretation. A longitudinal study design, which aims to investigate the effect of menopause status or aging on MFO rate, would be preferable, but this is difficult due to logistical reasons.

In the present study, we only measured plasma concentrations at rest, and it is important to note that the exercise‐induced change in substrates and metabolites—and in particular for plasma FFA—may differ with age and this could affect fat oxidation in skeletal muscles through exogenous fat delivery. Further studies are needed to investigate this.

In summary, we find that in trained women, the absolute MFO rate is higher in young compared to both middle‐aged and older women. Furthermore, middle‐aged women have a higher absolute MFO rate compared to older women, and this implies that it is age per se and the accompanying decreased V̇O_2max_, and not a change in plasma–estrogen exposure that leads to a lower absolute MFO rate. We further demonstrate that in trained women, FAT_max_ remains unchanged with aging. Further studies should address to what extent these age induced changes impact on metabolic flexibility across the lifespan.

## AUTHOR CONTRIBUTIONS

J. Frandsen, R. E. Sahl, and J. W. Helge designed the research. I. M. Dahlgaard Hansen, J. F. Wismann, M. Hansen, A. Ingersen, M. Schmücker, and J. L. Modvig performed the study. I. M. Dahlgaard Hansen, and J. F. Wismann performed the analyses. I. M. Dahlgaard Hansen, J. F. Wismann, and J. W. Helge wrote the manuscript, and all authors read, contributed and approved the final manuscript.

## CONFLICT OF INTEREST STATEMENT

The authors declare no conflict of interest.
